# Cytochrome P4504A inhibitors attenuate the exaggerated natriuretic response to volume expansion in thyroidectomized rats

**DOI:** 10.14814/phy2.12040

**Published:** 2014-06-11

**Authors:** Cecilia Colombero, Marcela Venara, Daniel Gonzalez, Richard J. Roman, Susana Nowicki

**Affiliations:** 1 Centro de Investigaciones Endocrinológicas “Dr. César Bergadá” (CEDIE), CONICET – FEI – División de Endocrinología, Hospital de Niños Ricardo Gutiérrez, Buenos Aires, Argentina; 2 Department of Pharmacology and Toxicology, The University of Mississippi Medical Center, Jackson, Mississippi

**Keywords:** 20‐HETE, cytochrome P450‐4A, eicosanoids, hypothyroidism, sodium excretion

## Abstract

Thyroidectomy augments the natriuretic response to volume expansion; however, the mechanism remains unknown. This study assessed the role of 20‐hydroxyeicosatetraenoic acid (20‐HETE) in the natriuretic response to an acute volume expansion in hypothyroid rats. Urine flow (1.9‐fold), sodium excretion (2.4‐fold), fractional sodium excretion (3.8‐fold), and distal delivery of sodium (4.1‐fold) increased to a greater extent in thyroidectomized rats (TX) than in sham‐operated controls (SHAM) following i.v. infusion of isotonic saline (5% body weight) over 60 min. This was associated with inhibition of both proximal and distal tubular reabsorption of sodium. Administration of two mechanistic and chemical dissimilar inhibitors of the synthesis of 20‐HETE, 1‐aminobenzotriazole (ABT), and N‐hydroxy‐N’‐(‐4‐butyl‐2‐methylphenyl)formamidine (HET0016) decreased the natriuretic response in TX rats. Glomerular filtration rate was lower in TX than in SHAM rats and was not altered by the CYP4A inhibitors. The expression, intrarenal distribution, and the formation of 20‐HETE and expoxygenase metabolites of arachidonic acid were similar in the cortex and medulla of SHAM and TX rats. These results suggest that CYP4A‐derived metabolites of arachidonic acid play an important role in the enhanced natriuretic response to volume expansion in hypothyroid rats even though TX did not alter the expression or activity of these enzymes.

## Abstract (precis)

Thyroidectomy increases the natriuretic response to volume expansion; however, the mechanism remains unknown. We tested the hypothesis that P‐450 metabolites of arachidonic acid contribute to the enhanced natriuretic response to an acute sodium load in hypothyroid rats. Our results indicate that inhibition of the formation of 20‐HETE (a CYP4A‐ arachidonic acid metabolite) blunts the enhanced natriuretic response to volume expansion in thyroidectomized animals by enhancing sodium transport in the distal nephron.

## Introduction

The excretion of sodium and water under basal conditions (Bradley et al. 1974) and the natriuretic response to volume expansion (Holmes and DiScala 1970; Singleton et al. 1999) is markedly elevated in thyroidectomized rats. However, the mechanism for the enhanced natriuretic response remains to be determined.

Cytochrome P450 (CYP) metabolites of arachidonic acid (AA) in the kidney play an important role in the renal tubular handling of salt and water (Webb et al. 1996; Capdevila et al. 2007; Fernandez et al. 2012). Arachidonic acid is metabolized in the kidney of rats by CYP enzymes of the 2C and 2J pathways to epoxyeicosatrienoic acids (EETs) and by CYP enzymes of the 4A family to 20‐hydroxyeicosatetraenoic acid (20‐HETE). EETs inhibit sodium transport in the proximal tubule and collecting duct (Pavlov et al. 2011) while 20‐HETE inhibits Na^+^/H^+^ exchange in the proximal tubule, Na^+^,K^+^‐2Cl^‐^ transport in the thick ascending limb of Henle's loop, and Na^+^,K^+^‐ATPase activity in both nephron segments (Webb et al. 1996; Nowicki et al. 1997; Yu et al. 2007).

Previous studies have demonstrated that acute volume expansion increases the renal release of dopamine (DA) and that DA contributes to the natriuretic response by inhibiting Na^+^,K^+^‐ATPase activity in both the proximal tubule and thick ascending loop of Henle (Aperia 2000). Moreover, inhibitors of the synthesis of 20‐HETE have been reported to blunt the natriuretic response to DA infusion (Fernandez et al. 2012), high‐salt diet (Hoagland et al. 2003), or acute elevations in blood pressure (Dos Santos et al. 2004; Williams et al. 2010).

In addition, other studies have indicated that the expression of CYP4A enzymes is regulated by the peroxisome proliferator‐activated alpha receptor (PPARα) (Johnson et al. 2002; Fernandez et al. 2012). Cooperative heterodimerization of PPARα to the retinoid X receptor (RXR) is obligatory for PPARα‐dependent gene transcription. In turn, RXR serves a similar role as a partner for the binding of the receptors for thyroid hormone (triiodothyronine, T3), retinoic acid, and vitamin D. Thus, the activity of other nuclear receptor signaling pathways that utilize RXR may modulate PPARα activity (Johnson et al. 1996). Indeed previous studies have indicated that hyperthyroid state in rats reduces the ability of the peroxisome proliferating agent dehydroepiandrosterone (DHEA) to increase the renal expression of CYP4A1 and CYP4A3 mRNA by 60–80%, and to completely inhibit the induction of CYP4A2. This suggests that thyroid status of the animal may affect the expression of CYP4A enzymes (Vargas et al. 2006; Schwarz et al. 2012).

Thus, the purpose of this study was to test the hypothesis that CYP4A metabolites of arachidonic acid contribute to the enhanced natriuretic response to an acute sodium load seen in hypothyroid rats.

## Methods

### General

Experiments were performed on 9‐ to 10‐week‐old male Sprague Dawley rats that were purchased from the Animal Breeding Facility from the School of Biochemistry, University of Buenos Aires. The rats were housed in an Animal Care Facility at the Ricardo Gutierrez Children′s Hospital and had free access to food and water throughout the study. All protocols were performed according to the guidelines recommended by the National Institutes of Health and were reviewed by the Institutional Animal Welfare Committee in Buenos Aires.

Six experimental groups were studied. Group 1 served as the control and the experiments were performed on euthyroid rats. In Group 2, the experiments were performed on hypothyroid rats (TX) that were subjected to thyroidectomy at 5 weeks of age and supplemented with 0.9% Ca_2_Cl in drinking water. Group 3 were sham‐operated control rats (SHAM) that received 0.9% Ca_2_Cl in drinking water. Group 4 were TX rats that received hormone replacement with triiodothyronine (TX + T3) 5 *μ*g/100 g for seven consecutive days prior to the experiment. Group 5 consisted of TX rats treated with the nonselective CYP4A inhibitor 1‐aminobenzotriazole (TX + ABT) 50 mg/kg, i.p., 18 h before the experiment. This dose has been shown to inhibit the formation of 20‐HETE by >80% while reducing formation of EETs and DiHetes by about 50% (Hoagland et al. 2003; Dos Santos et al. 2004). Group 6 were TX rats treated with a highly selective inhibitor of the synthesis of 20‐HETE, N‐hydroxy‐N’‐(‐4‐butyl‐2methylphenyl) formamidine (TX + HET0016) 10 mg/kg/day, i.p. for three consecutive days prior to the experiment. This dose has been reported to completely inhibit the formation of 20‐HETE and has no significant effect on epoxygenase activity (Hoagland et al. 2003; Williams et al. 2007). ABT was dissolved in isotonic saline, whereas HET0016 was dissolved in 3% mannitol + 11% captisol (sulfobutyl ether beta cyclodextrin). Neither of the vehicles had an effect on any of the studied parameters (data not shown).

Additional experiments were performed to confirm the results obtained in TX rats using a second hypothyroid model in rats treated with 6‐n‐propyl‐2‐thiouracil in water (PTU). Group 7 served as control, and the rats in Group 8 were treated with PTU (0.05 % P/V in drinking water for 3 weeks (PTU) (Mourouzis et al. 2009). Group 9 consisted of PTU rats treated with HET0016 10 mg/kg/day, i.p. for three consecutive days prior to the experiment (PTU + HET0016).

The thyroid state of the animals was confirmed before clearance studies by measuring serum T3 levels.

### Clearance studies

The night before the acute clearance study, rats from Groups 1, 2, and 3 were placed in metabolic cages to measure baseline urine flow, sodium, and water excretion. Then, the rats were anesthetized with thiopental (70 mg/kg, i.p.) and surgical prepared for a renal clearance experiment. The trachea was cannulated to facilitate breathing. Catheters were placed in the carotid artery and femoral vein to measure mean arterial pressure and for i.v. infusions. The rats received an i.v. infusion of 0.9% NaCl solution containing FITC‐labeled inulin (4 mg/mL) and LiCl (5 mmol/L) at a rate of 3 mL/h. After surgery and a 45‐min stabilization period, urine and blood samples (0.4 mL) were collected during two control periods of 15 min each. The animals then received an i.v. infusion of 5% of the rat's body weight of 0.9% NaCl solution over a 60‐min period. Urine flow and sodium excretion were measured during 6 sequential 15‐min clearance periods.

### Determination of the effect of thyroidectomy on the renal expression of CYP4A proteins

At the end of the clearance experiment the left kidney was collected, separated into cortex and medulla, frozen in liquid nitrogen, and stored at −80°C until it was processed for measurement of the expression of CYP4A enzymes. The other kidney was taken for immunohistochemistry as described below. Samples of the renal cortex and medulla were homogenized in a 10 mmol/L potassium buffer (pH 7.7) containing (in mmol/L) 250 sucrose, 1 EDTA, and 0.1 phenylmethylsulfonylfluoride (PMSF). The homogenate was centrifuged at 3000 *g* for 5 min and the supernatant was centrifuged at 11,000 *g* for 15 min. The protein concentration in the supernatant was measured using the Bradford method. Aliquots (10 *μ*g) were separated on a 10% SDS‐PAGE gel and transferred to PVDF membranes. Blots were probed with rabbit polyclonal antibody to CYP4A (1:1500, Abcam, Cambridge, U.K.). The membranes were stripped, washed, and reblotted with mouse anti‐*β*‐tubulin primary antibody (1:3000; SigmaAldrich Co, Saint Louis, MO). The blots were developed using ECL Plus Western Blotting detection system (GE Healthcare UK Limited, Little Chalfont, Buckinghamshire, U.K.). Detection and the relative intensity of the bands in the 50kDa were analyzed by HP Precision Scan software (Hewlett‐Packard, Palo Alto, CA). The results are expressed as the relative intensity of the CYP4A/*β*‐tubulin bands.

### Expression of CYP4A mRNA

The relative expression of CYP4A1, CYP4A2 and CYP4A3 mRNA, and *β*‐actin mRNA (internal standard for each sample) was determined in the cortex and medulla from SHAM and TX rats using PCR. Total RNA was extracted from renal cortex and medulla with Trizol^®^. One *μ*g of total RNA was reversed transcribed using SuperScript II^®^ and 1 *μ*L of the cDNA was used for PCR using primers published elsewhere (Ito et al. 1998). Optimal amplification conditions were obtained for each PCR. The structure of the primers used was as follows: P‐450 4A1, +5′ GTA TCC AAG TCA CAC TCT CCA 3′ and −5′ CAG GAC ACT GGA CAC TTT ATT G 3′; P‐450 4A2, +5′ AGA TCC AAA GCC TTA TCA ATC 3′ and −5′ CAG CCT TGG TGT AGG ACC T 3′; P‐450 4A3, +5′ CAA AGG CTT CTG GAA TTT ATC 3′ and −5′ CAG CCT TGG TGT AGG ACC T 3′; P‐450 4A8, +5′ GGG CAT GAG TGG CTC GG 3′ and −5′ GCA ATG ACC TGA GCT TTA TTC 3′; *β*‐actin, +5′ TAA AGA CCT CTA TGC CAA CA 3′ and −5′ GAG CCA CCG ATC CAC A 3′. For CYP4A1 and CYP4A3, the cycling conditions were 94°C for 1 min, 60°C for 2 min, and 72°C for 2 min for 27 cycles. The CYP4A2 reactions were cycled using a step‐down protocol for 94°C for 1 min, 70°C for 2 min, and 72°C for 2 min, for nine cycles followed by 94°C for 1 min, 60°C for 2 min, and 72°C for 2 min, for 27 cycles. The samples were separated on 2% agarose gels and stained with Ethidium Bromide.

### Immunohistochemistry

The right kidney was fixed by the retrograde perfusion through the abdominal aorta with 200 mL of 4% paraformaldehyde containing 0.2% picric acid in 0.1 mol/L phosphate buffer at 4°C. The kidneys were collected and immersed in 4% paraformaldehyde for 24 h. The kidneys were embedded in paraffin, and 5‐*μ*m‐thick sections were prepared. The antigens were retrieved by microwave‐oven treatment in sodium citrate buffer 0.01 mol/L, pH 6 for 3 min. The endogenous peroxidase activity was subsequently blocked with 3% H_2_O_2_ and the nonimmunologic binding with phosphate‐buffered saline containing 1% bovine serum albumin. The slides were incubated with a rabbit polyclonal antibody to CYP4A (1:500, Abcam) for 1 h at 4°C then extensively washed with TBS. The antibody complex was detected using the Sensitive Link‐Label IHC detection system and 3,3′‐diaminobenzidine (DAB) as chromogen (BioGenex, San Ramon, CA).

### CYP metabolism of Arachidonic Acid

One mg of renal cortical or medullary homogenate was incubated with a saturating concentration of arachidonic acid (40 *μ*mol/L) for 1 h in the presence of 1 mmol/L NADPH. The incubations were stopped by acidification to pH 3.5 with formic acid, homogenized, and the homogenate was extracted twice with 3 mL of ethyl acetate after the addition of 2 ng of an internal standard, d6‐20‐hydroxyeicosatetraenoic acid. The organic phase was collected and dried under nitrogen. The samples were reconstituted with 50% methanol and water, and the metabolites were analyzed using a Dionex (Sunnyvale, CA) HPLC system and an ABsciex 4000 Q trap tandem mass spectrometer with electrospray ionization (ABsciex, Foster City, CA) as described previously (Fan et al. 2013).

The metabolites were first separated using a reverse‐phase column (Beta basic C18, 150 × 2.1 mm, 3 *μ*m; Thermo Hypersil‐Keystone, Bellefonte, PA) and the following mobile phase conditions at a flow rate of 300 *μ*L/min. The column was equilibrated with 66.7% of a mobile phase A containing water/acetonitrile/methanol/acetic acid 90/8.5/1.4/0.1 (v/v/v/v), and 33.3% mobile phase B, acetonitrile/methanol 85/15 for 5 min following injection of the sample. The percentage of mobile phase B was ramped to 53.5% over a 10‐min period and then held there for 5 min followed by a linear increase to 94.4% mobile phase B over a 7‐min period and then held there for another 5 min. Column temperature was maintained at 35°C.

The products were ionized using the negative ion mode and using multiple reaction monitoring (MRM) with the following instrument settings: electrospray voltage −4500 volts, curtain gas 30, gas 1–50, temperature 600, gas 2–50, and unit resolution. The following transitions were monitored for each metabolite of AA; m/z 337‐207 (14,15‐DIHETE), m/z 337‐167 (11,12‐DIHETE), m/z 337‐127 (8,9‐DIHETE), m/z 319‐231 (19‐HETE), m/z 319‐245 (20‐HETE), m/z 319‐261 (18‐HETE), m/z337‐145 (5,6‐DIHETE), m/z 319‐233 (16‐HETE), m/z 319‐175 (15‐HETE), m/z 319‐149 (11‐HETE), m/z 319‐179 (12‐HETE), m/z 319‐155 (8‐HETE), m/z 319‐203 (5‐HETE), m/z 319‐175(14,15‐EET), m/z 319‐167 (11,12‐EET), m/z 319‐127 (8,9‐EET), m/z 319‐191 (5,6‐EET), and m/z 325‐281/307 (d6 20‐HETE) for the internal standard.

The ratios of ion abundance in the peaks of interest versus those seen in the internal standard were determined and compared with standard curves generated over a range from 0.1 to 2.0 ng for 20‐HETE and from 1.0 to 10 ng for the other metabolites. Values are expressed as picomoles of product formed per minute per milligram of protein. Epoxygenase activity was calculated as the sum of the products 5,6‐EET, 8,9‐EET, 11,12‐EET, 14,15‐EET and their stable metabolites 5,6‐ DIHETE, 8,9‐ DIHETE, 11,12‐ DIHETE, 14,15‐ DIHETE, respectively.

### Analysis of urine and plasma samples

The concentration of FITC inulin in plasma and urine samples was measured using a Synergy HT microplate reader (BioTek Instruments, Inc. Winooski, VT) with excitation and emission wavelengths of 480 and 530, respectively. Plasma T3 levels were measured using an electrochemiluminescence immunoassay (Roche Diagnostics, Cobas e411, Mannheim, Germany). Catecholamines (Dopamine and Noradrenaline) were extracted from urine using alumina, separated by reverse‐phase high‐pressure liquid chromatography using a 4.6 × 150 mm, 5 *μ*m C18 column (Agilent Life Sciences and Chemical Analysis, Santa Clara, CA), and quantified amperometrically using a triple‐electrode system (ESA, Bedford, MA) (Eisenhofer et al. 1986). Sodium concentration in plasma and urine was measured by flame photometry, and lithium levels were determined by Atomic Emission Spectrophotometry using a Varian 240 FS atomic absorption spectrometer (Agilent Technologies Inc., CA).

Urinary sodium excretion was calculated as (Urinary [Na^+^] × Urine output (V)). Glomerular filtration rate (GFR) was calculated from the clearance of FITC Inulin. The filtered load of sodium was calculated as (Plasma [Na^+^] × GFR), and fractional sodium excretion (FE Na^+^) as (CNa^+^/CIN × 100). Fractional delivery of sodium from the proximal tubule (FE Na^+^ prox) was calculated as (CLi^+^/CIN), and fractional delivery from the distal nephron (FE Na^+^ dist) was calculated as (CNa^+^/CLi^+^) (Christensen et al. 1986).

### Statistics

Data are presented as mean values ± SEM. The significance in differences in mean values was performed using a two‐way repeated measures ANOVA followed by a Bonferroni post hoc test. *P* value <0.05 was considered statistically significant.

## Results

### General

Table 1 presents the characterization of the control, sham‐operated (SHAM), and thyroidectomized (TX) rats (Groups 1, 2, and 3). Thyroidectomy reduced serum levels of T3, and kidney and body weight. Noradrenaline excretion, an indicator of sympathetic activity, was significantly greater in TX than SHAM rats. Food and water intake was not significantly different in the TX and SHAM rats. Similarly, 24‐h urine volume, sodium excretion, urinary osmolarity, and serum sodium concentrations were not significantly different in the TX versus the SHAM rats.

**Table 1.** Characteristics of study groups

	Control (*n* = 5)	SHAM (*n* = 5)	TX (*n* = 5)	SHAM versus TX	Control versus SHAM
Free T3, ng/dL	121.2 ± 6.0	129.2 ± 2.6	56.1 ± 4.9	*P* < 0.001	N.S.
Body weight, g	271 ± 17	226 ± 13	158 ± 11	*P* < 0.01	N.S.
Daily body weight gain, %	4.85 ± 0.12	4.24 ± 0.32	1.52 ± 0.17	*P* < 0.001	N.S.
Kidney weight, g	2.30 ± 0.28	1.88 ± 0.13	1.28 ± 0.09	*P* < 0.05	N.S.
Food intake, g/24 h	14.2 ± 1.6	13.7 ± 1.6	11.7 ± 1.3	N.S.	N.S.
Water intake, mL/24 h	23.9 ± 5.3	31.2 ± 5.2	23.8 ± 6.1	N.S.	N.S.
Diuresis, mL/24 h 100 g	5.13 ± 0.80	7.89 ± 1.71	7.63 ± 2.05	N.S.	N.S.
Natriuresis, mEq/24 h 100 g	0.67 ± 0.06	0.70 ± 0.07	0.59 ± 0.17	N.S.	N.S.
Urine Osmolarity, mOsm/L	1982 ± 103	1523 ± 146	1518 ± 213	N.S.	N.S.
Plasma Na^+^, mEq/L	139.0 ± 1.5	139.2 ± 2.0	138.2 ± 3.0	N.S.	N.S.
NA excretion, ng/24 h 100 g	428 ± 127	411 ± 17	1387 ± 287	*P* < 0.05	N.S.

Values are means ± SE. TX, thyroidectomized rats; T3 triiodothyronine; NA, noradrenaline.

### Comparison of the response to volume expansion

**Figure 1.**
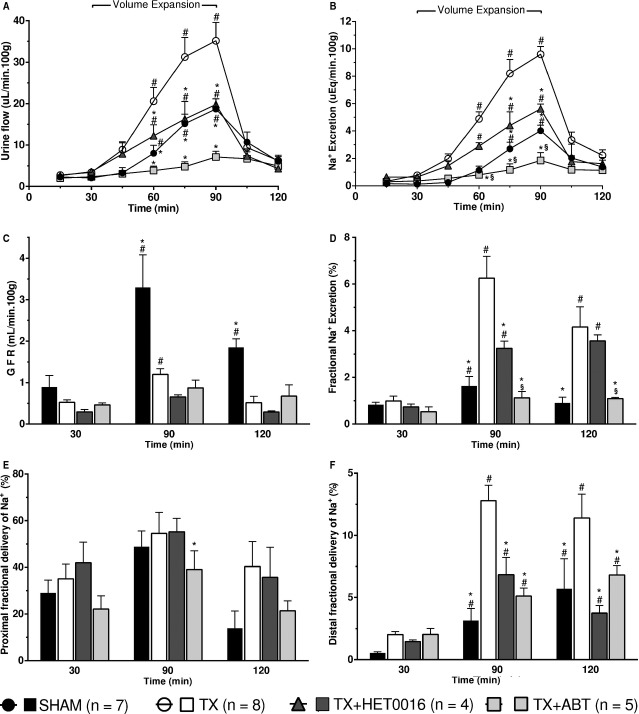
Comparison of the effects of 1‐aminobenzotriazole (ABT, 50 mg/kg, i.p.) and N‐hydroxy‐N’‐(‐4‐butyl‐2methylphenyl) formamidine (HET0016, 10 mg/kg/day, i.p. 3 days) on the natriuretic response to i.v. infusion of isotonic saline (5% body weight, 60 min) in thyroidectomized (TX) rats. Numbers in parentheses indicate the number of animals studied per group. Values are means ± SEM. *indicates *P *<**0.05 versus TX rats; ^#^*P *<**0.05 versus the value prior to the volume expansion (30 min); ^§^*P *<**0.05 TX + ABT versus TX + HET0016.

Figure 1 compares the response to volume expansion in SHAM, TX, TX + ABT, and TX + HET0016 rats (Groups 2, 3, 5, and 6). Baseline urine flow was similar in all the groups. After volume expansion, urine flow increased to a greater extent in TX than in the SHAM controls (35.2 ± 4.5 vs. 18.7 ± 2.5 *μ*L/min 100 g; *P* < 0.05). Treatment of TX rats with ABT completely abolished the increase in urine flow in TX rats (7.1 ± 1.4 *μ*L/min 100 g; *P* < 0.05 vs. TX rats). Similarly, HET0016 markedly reduced the increase in urine flow in the TX‐treated animals (19.7 ± 1.4 *μ*L/min 100 g; *P* < 0.05 vs. TX rats) (Fig. 1A).

The effects of TX and the CYP inhibitors on the natriuretic response to volume expansion followed a similar pattern. Baseline sodium excretion was similar in SHAM and TX rats. Sodium excretion increased to a greater extent in TX animals than in the SHAM controls (9.6 ± 0.5 vs. 4.0 ± 0.4 *μ*Eq/min 100 g; *P* < 0.05). The natriuretic response to volume expansion in the TX animals was completely blocked by ABT (2.0 ± 0.6 *μ*Eq/min 100 g; *P* < 0.05 vs. TX rats) and was reduced by 42% in TX rats treated with HET0016 (5.6 ± 0.4 *μ*Eq/min 100 g; *P* < 0.05 vs. TX rats) (Fig. 1B).

There were no significant differences in GFR under basal conditions between the various treatment groups. GFR increased to a greater extent in SHAM than in TX animals after volume expansion (3.2 ± 0.3 vs. TX 1.2 ± 0.2 mL/min 100 g; *P* < 0.05). Inhibition of CYP activity with either ABT or HET0016 had no significant effect on GFR before or after volume expansion in TX rats (Fig. 1C). The filtered load of sodium was lower in TX than in SHAM rats during volume expansion (126 ± 20 vs. 576 ± 175 *μ*Eq/min; *P* < 0.01) and it was not modified by treatment of TX rats with inhibitors of CYP activity.

Baseline fractional excretion of sodium was similar in TX and SHAM rats. After volume expansion it increased to a far greater extent in TX animals than in SHAM control rats (6.2 ± 0.9 vs. 1.6 ± 0.3%; *P* < 0.05). ABT completely blocked the increase in the fractional excretion of sodium after volume expansion in the TX rats (1.1 ± 0.3%; *P* < 0.05 vs. TX rats). HET0016 attenuated the increase in fractional sodium excretion by about 50% in these animals (3.2 ± 0.3%; *P* < 0.05 vs. TX rats) (Fig. 1D).

The fractional excretion of lithium was used to estimate the inhibition of sodium transport in the proximal and distal nephron after volume expansion. Baseline fractional excretion of lithium, an index of proximal tubular reabsorption, was similar in all the groups. It increased in all the groups after volume expansion; however, it was significantly lower in TX rats treated with ABT than in the TX group (TX + ABT 39 ± 8 vs. 54 ± 9% in TX; *P* < 0.05) (Fig. 1E). The fractional delivery of sodium from the distal nephron increased to a far greater extent in TX rats after volume expansion than in the SHAM control animals (TX 12.8 ± 1.2 vs. 3.1 ± 0.9% in SHAM; *P* < 0.05). The greater inhibition of the sodium transport in the distal nephron was normalized in TX rats pretreated with HET0016 or ABT (6.8 ± 1.4 and 5.1 ± 0.6%, respectively; *P* < 0.05 vs. TX rats) (Fig. 1F).

Basal blood pressure was similar in SHAM and TX rats (142 ± 5 vs. 138 ± 3 mm Hg) and was not significantly altered during the experiment. Similarly, blood pressure was similar in ABT and HET0016‐treated rats versus the levels seen in TX rats.

**Figure 2.**
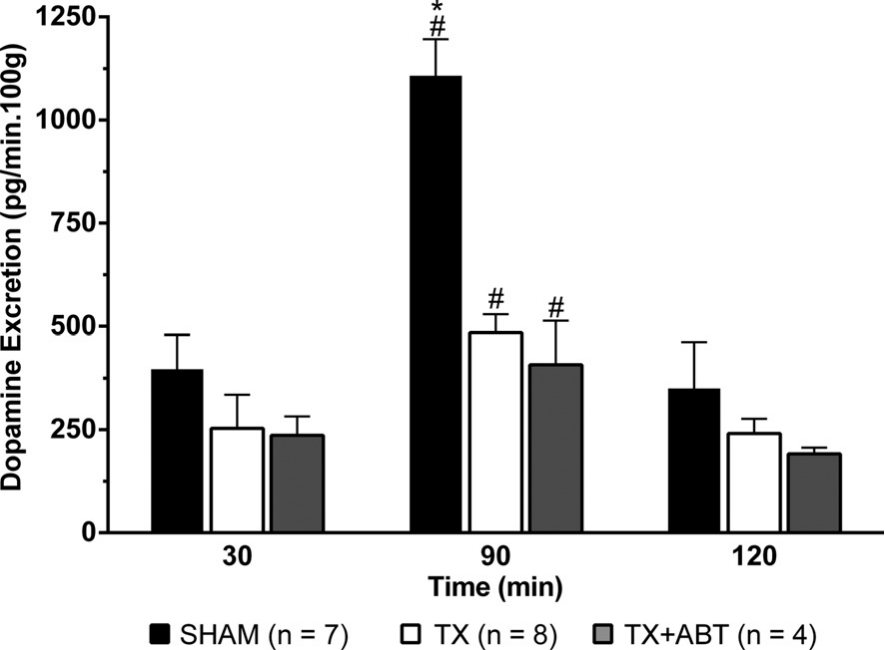
Effects of blockade of the renal CYP4A with 1‐aminobenzotriazole (ABT, 50 mg/kg, i.p.) on dopamine excretion in thyroidectomized (TX) rats during a control period (30 min) and after volume expansion with isotonic saline. Values are means ± SEM. *indicates *P *<**0.05 versus the corresponding value in TX rats; ^#^*P *<**0.05 versus the value prior to the volume expansion (30 min).

Dopamine excretion rate was not statistically different among SHAM, TX rats, and TX rats treated with ABT during the control period. Dopamine excretion increased after saline infusion in all groups. Treatment with ABT did not modify DA excretion in response to volume expansion in TX rats (Fig. 2). Similarly, there was no significant difference in noradrenaline excretion between TX rats and TX rats treated with ABT (data not shown).

The effect of thyroidectomy to elevate the sodium excretion was prevented by T3 replacement in Group 4. As presented in Table 2, urine flow, GFR and sodium excretion were similar before and after volume expansion in CONTROL, SHAM, and TX + T3 rats (Groups 1, 3, and 4).

**Table 2.** Data on renal function at basal conditions and after 60 min of 5% body weight saline volume expansion in anesthetized rats

	FreeT3 (ng/dL)	Diuresis (*μ*L/min 100 g)		Natriuresis (*μ*Eq/min 100 g)		Glomerular filtration rate (mL/min 100 g)		Fractional sodium excretion (%)	
		Basal	Expansion	Basal	Expansion	Basal	Expansion	Basal	Expansion
Control (*n* = 5)	121.2 ± 6.0	1.85 ± 0.17	15.6 ± 1.76	0.11 ± 0.03	4.71 ± 0.64	0.85 ± 0.13	2.15 ± 0.34	0.12 ± 0.05	2.62 ± 0.18
SHAM (*n* = 7)	120.8 ± 3.4	2.08 ± 0.13	18.70 ± 2.46	0.11 ± 0.02	4.00 ± 0.39	0.89 ± 0.28	3.29 ± 0.79	0.18 ± 0.07	1.83 ± 0.48
TX + T3 (*n* = 5)	104.3 ± 9.8	2.37 ± 0.85	14.73 ± 2.55	0.18 ± 0.12	4.46 ± 1.05	0.73 ± 0.05	2.88 ± 0.36	0.15 ± 0.11	1.98 ± 0.57

Values are means ± SE. TX + T3, thyroidectomized rats replaced with triiodothyronine.

**Figure 3.**
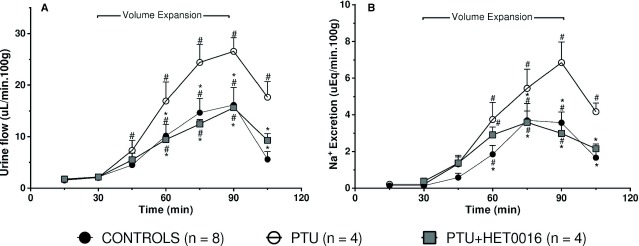
Effect of N‐hydroxy‐N’‐(‐4‐butyl‐2methylphenyl) formamidine (HET0016, 10 mg/kg/day, i.p. 3 days) on the natriuretic response to an i.v. infusion of isotonic saline (5% body weight, 60 min) in rats rendered hypothyroid by administration of 0.05 % P/V 6n‐propyl‐2‐thiouracil in water for 3 weeks (PTU). Numbers in parentheses indicate the number of animals studied per group. Values are means ± SEM. *indicates *P *<**0.05 versus the value prior to the volume expansion (30 min); ^#^*P *<**0.05 versus the corresponding value in control rats.

The effects of the CYP4A inhibitors to normalize the renal handling of sodium in a thyroid deficient state were confirmed by analyzing sodium excretion in control rats and rats treated with propylthiouracil (PTU) (Groups 7 and 8, respectively). Treatment with PTU decreased free serum T3 levels from 121 ± 6 to 72 ± 2 ng/dL (*P* < 0.01). Urine excretion during volume expansion was higher in PTU‐treated than in control rats (26.5 ± 2.6 vs. 16.1 ± 3.4 *μ*L/min 100 g; *P* < 0.05). Similarly, sodium excretion was significantly greater in PTU‐treated rats in comparison to the control group (6.8 ± 1.1 vs. 3.5 ± 0.6 *μ*Eq/min 100 g; *P* < 0.05). Treatment of the PTU‐hypothyroid rats with HET0016 (Group 9) restored both diuresis and natriuresis to values similar to controls (diuresis 15.6 ± 0.7 *μ*L/min 100 g; sodium excretion 4.0 ± 0.6 *μ*Eq/min 100 g; *P* < 0.05 vs. PTU for both) (Fig. 3).

### CYP4A expression and activity

**Figure 4.**
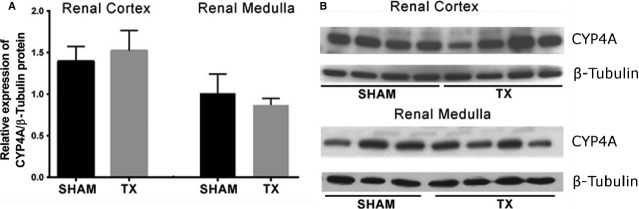
Expression of CYP4A and *β*‐Tubulin protein in homogenates of renal cortex and medulla prepared from SHAM or thyroidectomized (TX) rats. The densitometric analysis of the immunoreactivity of CYP4A, expressed as the ratio CYP4A/*β*‐Tubulin, of 3–4 animals/group is also shown. Values are means ± SEM.

**Figure 5.**
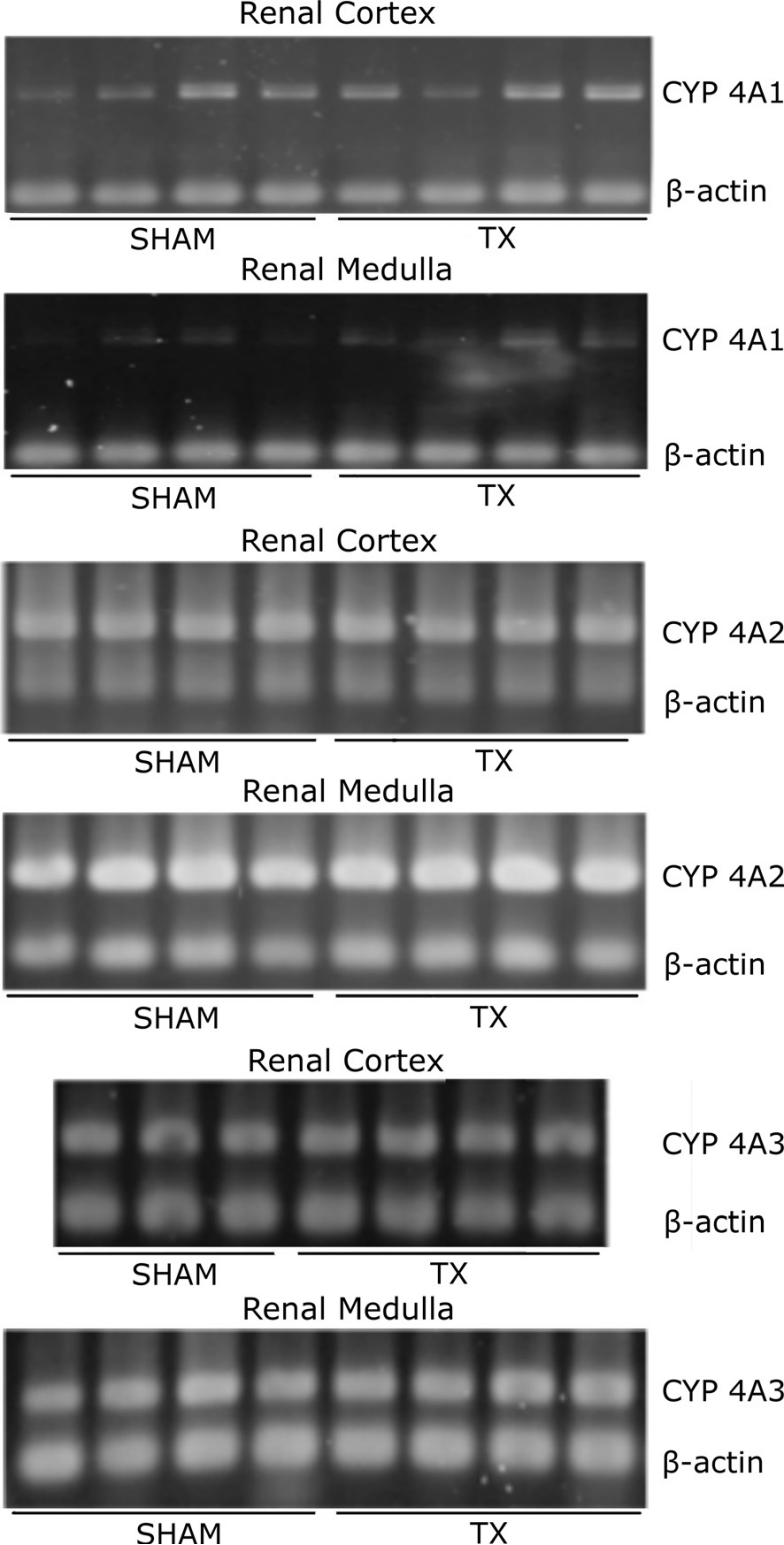
Expression of CYP4A mRNA in renal cortical and medullary homogenates prepared from SHAM or thyroidectomized (TX) rats (3–4 animals/group). Total RNA was isolated from tissue homogenates and CYP4A1, CYP4A2, CYP4A3 and *β*‐actin gene expressions were determined by RT‐PCR.

The expression of the CYP4A protein in the renal cortex and medulla isolated from SHAM and TX rats (Groups 2 and 3) is presented in Figure 4. CYP4A expression was higher in cortex than in medulla, but it was not different between SHAM and TX rats. Similarly, no differences were found in the expression of CYP4A mRNA levels in cortex or medulla isolated from TX versus SHAM rats (Fig. 5).

**Figure 6.**
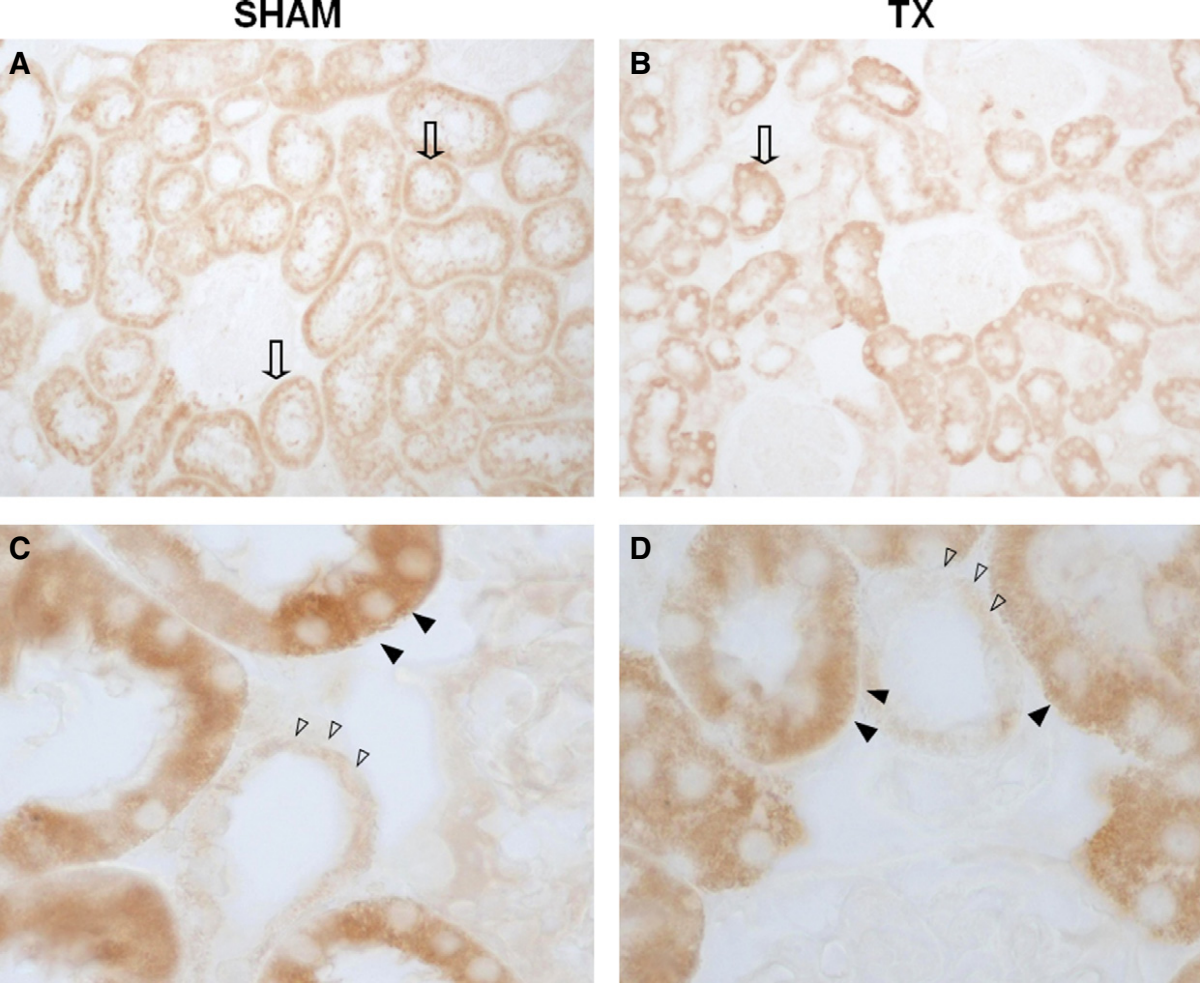
CYP4A distribution in cortical sections: Positive staining is observed in the cytoplasm of tubular cells (empty arrows) in both SHAM (Panel A, B) and TX rats (Panel C, D). Staining is more intense in proximal tubule cells (arrowheads) than in thick ascending loop of Henle cells (empty arrowheads).

CYP4A protein was expressed to a greater extent in the proximal tubule than in the thick ascending loop of Henle, but there was no obvious difference in the intensity or distribution of CYP4A staining in the sections obtained from SHAM and TX rats (Fig. 6).

We also examined the production of 20‐HETE and epoxygenase metabolites (EETs and DiHETEs) in the renal cortex and outer medulla of SHAM and TX rats incubated with arachidonic acid. The results presented in Table 3 indicate that the production of 20‐HETE was higher in cortex than in medulla, but there was no difference in the rate of production of 20‐HETE or Epoxygenase metabolites of AA in SHAM and TX rats.

**Table 3.** CYP activity

	ng/0.5 mg protein 60 min			
	20‐HETE		Epoxygenase products	
	SHAM	TX	SHAM	TX
Renal cortex	284 ± 23	214 ± 15	127 ± 13	97 ± 13
Renal medulla	36 ± 4	34 ± 8	89 ± 12	63 ± 18

Values are means ± SE. TX thyroidectomized rats. Epoxygenase activity was calculated as the sum of the products 5,6‐EET, 8,9‐EET, 11,12‐EET, 14,15‐EET, and their stable metabolites 5,6‐ DIHETE, 8,9‐ DIHETE, 11,12‐ DIHETE, 14,15‐ DIHETE, respectively.

## Discussion

The results of this investigation suggest that CYP4A metabolites of arachidonic acid play an important role in the enhanced natriuretic response to volume expansion in hypothyroid rats. This conclusion is based on the observation that administration of two chemically and mechanistically different inhibitors of the renal formation of 20‐HETE reduced the diuretic and natriuretic response to an acute volume expansion in TX rats. The present finding that ABT, which blocks the formation of both EETs and 20‐HETE, reduced the natriuretic response more than that seen using the specific 20‐HETE inhibitor, HET0016, suggests that EETs may also contribute to the enhanced natriuretic response to volume expansion in TX rats.

Advanced hypothyroidism is associated with significant abnormalities of renal function, sodium wasting and hyponatremia, an impaired ability to concentrate urine, and a reduction in GFR associated with increased sympathetic activity (Vargas et al. 1994; Iglesias and Díez 2009). In this study, using a moderate model of hypothyroidism in which free T3 levels were reduced only by 50%, body and kidney weight was reduced and urinary noradrenaline excretion was elevated as expected. However, in line with previous findings in hypothyroid rats (Taylor and Fregly 1964; Cadnapaphornchai et al. 2003) and patients (Baajafer et al. 1999; Asami and Uchiyama 2004; Schwarz et al. 2012), the rats still were able to remain in sodium balance and baseline urine flow and sodium excretion was not significantly different in the SHAM‐operated rats and TX rats.

Early studies performed on rats rendered hypothyroid by thyroidectomy or chemical means showed that hypothyroid rats exhibit an increased urine output and fractional excretion of sodium after saline loading (Holmes and DiScala 1970; Michael et al. 1972). The present findings are consistent with these previous results. Urine flow and sodium excretion increased to a far greater extent in TX rats than in SHAM controls after acute volume expansion in our study. Similar results were obtained in a second PTU‐induced model of hypothyroidism. Moreover, the enhanced natriuretic response to volume expansion in TX animals was reversed by T3 replacement. These findings exclude the possibility that augmented response in TX rats was secondary to electrolyte disturbances or due to damage to the parathyroid glands during surgery.

It is well known that hypothyroidism is associated with a reduction in GFR that may be secondary to suppression of the Renin‐Angiotensin Aldosterone System (RAAS) (Vargas et al. 2006; Iglesias and Díez 2009). As expected, the increase in GFR triggered by volume expansion was less in TX than in SHAM rats. Further analysis of our results suggests that the increase in sodium excretion despite reduced GFR following volume expansion in TX rats was largely due to a greater inhibition of tubular reabsorption. Inhibition of tubular transport in TX rats has been previously documented and has been attributed to the decreased expression of sodium transporters in hypothyroid rats (Schmitt et al. 2003; Mariani and Berns 2012; Moreno et al. 2012). The results of our lithium clearance study indicated that the enhanced natriuretic response after volume expansion was due to more marked inhibition of sodium transport in the distal nephron after volume expansion in the TX animals.

The fact that the increase in diuresis and tubular sodium excretion following saline infusion in TX rats was markedly attenuated by the inhibition of CYP4A activity strongly suggested that elevated levels of 20‐HETE contribute to the greater inhibition of tubular sodium reabsorption in hypothyroid rats. Based on previous reports that the expression of CYP4A is inhibited by thyroid hormones (Webb et al. 1996; Singleton et al. 1999), and on the present observation of the important contribution of 20‐HETE to the increased tubular sodium excretion in hypothyroid rats, we expected that CYP4A expression and 20‐HETE production would be greater in kidneys from TX rats than controls. However, the expression of CYP4A protein and the production of 20‐HETE or epoxygenase metabolites were not different in TX and SHAM rats. Moreover, the immunohistochemistry indicated that the intrarenal distribution of CYP4A was similar in TX and SHAM rats.

Nevertheless, in this study performed on TX rats, ABT reduced urine flow by 80%, sodium excretion by 81%, FENa^+^ by 82%, proximal FENa^+^ by 28%, and distal FENa^+^ by 61%. These findings are consistent with the results of a previous study in normal rats in which administration of ABT reduced the diuretic and natriuretic response to volume expansion by 60–70% (Fernandez et al. 2012). Similarly, our present result show that inhibition of the synthesis of 20‐HETE by HET0016 reduced sodium excretion after volume expansion and enhanced reabsorption in the distal nephron similar to ABT. However, the blunting of the response was greater in ABT than in HET0016‐treated rats because ABT but not HET0016 also enhanced sodium transport in the proximal tubule. These findings are consistent with the view that ABT also inhibits the inhibitory effects of EETs on sodium transport in the proximal tubule.

Neither of these inhibitors modified GFR values in TX rats suggesting that neither EETs nor 20‐HETE contribute to the decreased GFR in hypothyroid rats. Thus, CYP450 metabolites of arachidonic acid play an important role in the exaggerated natriuretic response to an acute volume expansion in hypothyroid rats and this is due largely to inhibition of sodium transport in the distal nephron. Indeed fractional delivery of sodium from the distal nephron was four times higher in TX than in SHAM rats after volume expansion. Inhibition of the formation of 20‐HETE with either HET0016 or ABT reduced fractional delivery of sodium from the distal nephron to the same level seen in the SHAM control animals. The mechanism involved remains to be determined. Inhibitors of the formation of 20‐HETE have been reported to enhance sodium transport in the thick ascending loop of Henle (Zou et al. 1996; Ito and Roman 1999), whereas inhibition of EETs enhance epithelial sodium channel (ENaC) activity and sodium transport in the cortical collecting duct (Pavlov et al. 2011).

It is well known that the increase in endogenously produced renal DA also contributes to the inhibition of sodium reabsorption following volume expansion (Hegde et al. 1989) and we have recently demonstrated that 20‐HETE plays a critical role in the natriuretic response to locally synthesized DA (Fernandez et al. 2012). The fact that in this study DA excretion during volume expansion was lower in TX than in SHAM rats rules out the possibility that the enhanced natriuretic response to volume expansion in TX rats was due to the increased intrarenal formation of DA.

In summary, the results of this study indicate that inhibition of the formation of 20‐HETE blunts the enhanced natriuretic response to volume expansion in thyroidectomized animals by enhancing sodium transport in the distal nephron. However, TX does not increase the expression of CYP4A enzymes or the renal production of 20‐HETE or EETs. Since inhibitors of 20‐HETE and EETs enhance sodium transport in TAHL and distal nephron and blunt the natriuretic response to volume expansion, it is possible that the effects of the CYP inhibitors reflect a physiological antagonism of the response to volume expansion in TX rats, that is, inhibition of the formation of 20‐HETE enhances sodium transport at the tubular level.

## Acknowledgments

The authors wish to thank Eduardo Dascal and Ana Uceda for their excellent technical assistance.

## Conflict of Interest

None declared.
